# Nuclear error phenotypes at the first mitosis predict cleavage-stage outcomes but not blastocyst competence

**DOI:** 10.3389/frph.2026.1905720

**Published:** 2026-08-06

**Authors:** Emma Adolfsson, Amanda Stenberg, Juliane Baumgart

**Affiliations:** 1 Department of Obstetrics and Gynecology, Faculty of Medicine and Health, Örebro University, Örebro, Sweden; 2 Department of Obstetrics and Gynecology, Örebro University Hospital, Örebro, Sweden

**Keywords:** assisted reproduction technology, embryo development, live birth predictors, nuclear error phenotypes, time-lapse imaging

## Abstract

**Background:**

Nuclear errors (NE) observed at the two-cell stage reflect early mitotic disturbances, but their clinical relevance for embryo implantation and live birth remains un-clear. We investigated whether nuclear status at the two-cell stage predicts pregnancy and live birth outcomes following cleavage-stage and blastocyst transfer.

**Methods:**

This retrospective cohort study included 3,793 single embryo transfers with clinical outcomes performed at a university-affiliated reproductive medicine centre. Embryos were retrospectively assessed at the two-cell stage and classified as normal or exhibiting nuclear errors, further stratified by the number of affected blastomeres and by phenotype (multinucleation, micronucleation, split nuclei, or mixed errors). Live birth rate (LBR) was the primary outcome; pregnancy and clinical pregnancy rates were secondary outcomes. Associations were evaluated using contingency analyses, regression models of nuclear error occurrence, and mixed-effects logistic regression analyses of clinical outcomes adjusted for maternal age, paternal age, fertilisation method, and patient-level clustering.

**Results:**

NE occurred in 24% of transferred embryos and were more frequent after ICSI than IVF (adjusted OR 1.74). In cleavage-stage transfers, NE were associated with reduced LBR compared with embryos without NE (23.2% vs. 34.8%). The clinical outcomes decreased with increased numbers of affected blastomeres. Micronucleation and split nuclei showed the poorest outcomes and remained adversely associated with reproductive outcomes after adjustment, whereas multinucleation was not detrimental. No association between NE and outcomes was observed after blastocyst transfers.

**Conclusions:**

Nuclear error phenotypes at the two-cell stage provided prognostic information relevant for cleavage-stage embryo transfer but had limited influence for blastocyst selection. Outcome differences associated with early nuclear abnormalities were no longer detectable among transferred blastocysts.

## 1 Introduction

Human reproduction as such is inefficient. Only approximately one in three fertilised oocytes results in a live birth, with even lower success rates following assisted reproductive technology (ART) treatments (1–5). In clinical settings where preimplantation genetic testing for aneuploidy (PGT-A) is restricted or prohibited, embryo selection relies solely on non-invasive morphological and morphokinetic assessment. Under these conditions, implantation rates for transferred blastocysts rarely exceed 50% (6), underscoring the need for improved non-invasive markers of embryonic competence.

The first mitotic division, which generates the two-cell embryo, represents a particularly vulnerable phase of human development and is highly error-prone (7). This division affects both daughter blastomeres and establishes the cellular foundation for all subsequent development. The first mitosis occurs approximately 27 h post-insemination, typically outside laboratory working hours. By the time embryos are assessed the following morning, most have completed the second mitotic division and reached the four-cell stage. As a consequence, direct evaluation of the two-cell stage in routine clinical practice has historically been limited.

The introduction of time-lapse imaging technology (TLT) has fundamentally altered this landscape, enabling uninterrupted monitoring of early embryonic development without disturbance. TLT permits detailed characterisation of events that are commonly missed by static observation, including nuclear dynamics during the first mitotic division and the two-cell stage. At the two-cell stage, nuclei appear as distinct, well-defined circular structures that form immediately after the first mitosis and persist until the second di-vision, after which assessment becomes increasingly challenging as development proceeds and cells become smaller.

Historically, abnormal nuclear morphology has been described using the broad term “multinucleation,” referring to blastomeres containing more than one visible nucleus. Multinucleation at the four-cell stage has been extensively studied and is associated with impaired embryo development, increased chromosomal abnormalities, implantation failure, and pregnancy loss (8–10). Time-lapse studies have demonstrated that multinucleation is both more prevalent and more dynamic than previously appreciated (11–13), prompting renewed interest in its origins and consequences. A recent review by Coticchio et al. summarises the current understanding of multinucleation at the two-cell stage, highlighting its complex aetiology and variable clinical implications (14).

Importantly, accumulating evidence indicates that multinucleation at the two-cell stage is not a uniform phenomenon. While several studies have reported associations between multinucleation and impaired embryo development, implantation, and live birth rates (15–17), others have shown that specific nuclear configurations—such as binucleation—may be compatible with normal development and favorable reproductive outcomes (18). These discrepancies have led to the introduction of the concept of nuclear error phenotypes (NEPs), which stratify abnormal nuclear patterns into biologically distinct categories that are likely to arise from different mitotic mechanisms and to carry different developmental consequences.

NEPs include binucleation, multinucleation, micronucleation, split nucleation, and mixed errors involving different phenotypes in the two blastomeres. Binucleation, characterised by two equally sized nuclei within a single cell, may result from dual spindle formation during the zygote-to-embryo transition and can be compatible with diploid genome segregation (19, 20). Micronucleation reflects the presence of small, additional nuclei and is associated with chromosome mis-segregation, lagging chromosomes, and genome instability (21–24). Multinucleation, defined here as the presence of three or more similarly sized nuclei, is thought to arise from aberrant spindle organisation or multipolar divisions (25, 26). Split nucleation represents a severe disruption of nuclear integrity, with fragmented nuclear material dispersed throughout the cytoplasm, whereas mixed errors describe embryos exhibiting different NEPs in each blastomere.

Recent mechanistic studies using advanced live-cell imaging have provided important biological context for these phenotype-specific nuclear abnormalities. Detailed analyses of the first mitotic division have demonstrated that spindle geometry, spindle bi-polarity, and chromosome congression directly influence the formation of multinucleated, micronucleated, and fragmented nuclear patterns in human embryos (7, 25). In addition, lineage-tracing studies have shown that the two blastomeres formed at the first cleavage contribute unequally to later embryonic lineages, indicating that early mitotic perturbations can have long-lasting developmental consequences (27). Together, these findings support the concept that NEP arise through distinct biological mechanisms and may differ meaningfully in their impact on subsequent embryo development and clinical outcome.

Despite growing recognition of NEPs, several critical questions remain unresolved. Nuclear errors identified at the two-cell stage represent transient historical events rather than persistent morphological features, raising uncertainty regarding their relevance for embryos developing to later stages and selected for transfer based on blastocyst morphology. Moreover, while several studies have examined implantation or pregnancy outcomes associated with early nuclear abnormalities (17, 28–31), comparatively little is known about phenotype-specific effects across developmental stages, and in particular their relevance for blastocyst quality at transfer and for live birth following single embryo transfer.

Building on this framework, in a previous study, we mapped nuclear error phenotypes at the two-cell stage using time-lapse imaging and demonstrated that these phenotypes differ markedly in their association with blastocyst formation rate (32). While binucleated embryos showed developmental potential comparable to embryos with normal nuclear morphology, other phenotypes—particularly split nucleation—were associated with substantially reduced likelihood of forming good-quality blastocysts. Furthermore, both the specific NEPs and the number of affected blastomeres influenced developmental competence, indicating biologically meaningful heterogeneity within the broader category of nuclear errors. However, that analysis was limited to preimplantation development. The phenotype-specific implications for blastocyst quality at transfer stage and for clinical outcomes, including live birth following single embryo transfer, remain unclear.

The aim of the present study was therefore to systematically characterise NEPs at the two-cell stage in a large ART cohort and to evaluate their phenotype-specific associations with clinically relevant outcomes, including live birth following single embryo transfer. By integrating time-lapse-based nuclear annotation with stage-specific clinical outcome data, we sought to determine which NEPs represent true biological liabilities and which may be mitigated by extended culture and morphology-based embryo selection.

## 2 Materials and methods

### 2.1 Study cohort

This non-interventional retrospective cohort study was conducted at the Reproductive Medicine Centre at the University Hospital of Örebro, Sweden.

The study included single embryo transfer cycles from 2012 to 2025 with complete clinical outcome data. Inclusion criteria were normal fertilisation defined as the presence of two pronuclei (2PN), and availability of complete time-lapse imaging data from fertilisation until embryo transfer.

Exclusion criteria comprised abnormal fertilisation, presence of irregular divisions or endomitosis, double embryo transfers, and incomplete clinical outcome data.

### 2.2 Assisted reproduction

Patients underwent controlled ovarian stimulation using either a gonadotropin-releasing hormone (GnRH) agonist protocol with mid-luteal down-regulation or a GnRH antagonist protocol. Oocyte retrieval was performed 36 h after ovulation triggering with either human chorionic gonadotropin or a GnRH agonist, using transvaginal ultrasound-guided follicular aspiration.

Spermatozoa were obtained from ejaculate samples and processed by either density-gradient centrifugation or motility-based selection chambers, depending on sperm quality. The fertilisation method was selected based on semen parameters and/or outcomes of previous ART cycles. Intracytoplasmic sperm injection (ICSI) was performed approximately four hours after oocyte retrieval, while conventional IVF was conducted using standard co-incubation procedures.

All embryos were cultured in a time-lapse incubator (EmbryoScope or EmbryoScope+, Vitrolife, Sweden) at 37  °C in an atmosphere of 6% CO_2_ and 5% O_2_, using pre-equilibrated culture media (sequential G-series G1/G2™ or G-TL™, Vitrolife, Sweden) under oil overlay (OVOIL™, Vitrolife, Sweden). Images were acquired every 15 min in multiple focal planes from fertilisation until embryo transfer and stored on a local server. The EmbryoScope and the EmbryoScope+ differ in the number of captured focal planes, 7 or 11, respectively.

Embryos were assessed daily based on conventional morphological criteria. Embryos were transferred either in fresh cycles or after vitrification in frozen replacement cycles, depending on embryo number and developmental progression. In fresh cycles, when five or more normally fertilised (2PN) zygotes were available, embryos were routinely cultured to the blastocyst stage. When four or fewer 2PN zygotes were obtained, embryo transfer was performed at the cleavage stage on day 2 or day 3, depending on the day of oocyte retrieval.

For day 2 transfers, embryos were required to contain at least two cells, preferably four cells, with equal blastomere size and minimal fragmentation [44 h post-insemination (HPI)]. For day 3 transfers, embryos were required to contain at least six cells, preferably eight, with equal blastomere size (66 HPI). In all cases, the best available embryo was selected for elective single embryo transfer. Embryos not selected for cleavage-stage transfer were cultured to the blastocyst stage.

For fresh day 5 transfers, blastocysts were assessed at 114 HPI and graded according to the Gardner and Schoolcraft grading system (33). The best available blastocyst was selected for transfer.

All remaining blastocysts graded ≥ 3BB were vitrified either on day 5 or day 6. In frozen replacement cycles, vitrified blastocysts were warmed at least two hours prior to transfer, and re-expansion was confirmed before transfer.

Embryo transfer was performed under ultrasound guidance using a soft tip transfer catheter. Luteal-phase support was administered according to standard clinical protocols. Pregnancy testing was performed using a home urine test 18 days after oocyte pick up/ ovulation. In cases of a positive result (hCG > 25 ng/mL), a transvaginal ultrasound ex-amination was conducted at gestational week 8–9 to confirm pregnancy location, number of amniotic sacs and fetuses, and fetal viability. Following delivery, patients reported pregnancy outcome, birthweight, neonatal sex, and neonatal health.

### 2.3 Assessment of nuclear status at the two-cell stage

Assessment of nuclear status was done as previously described (32). In each cell, the nuclear phenotype was classified as either mononucleated, binucleated, micronucleated or having split nuclei. Based on this, each embryo was categorized as either normal (mono-nucleated or binucleated) or having a nuclear error (NE). NEs were categorized as affecting one cell or two cells, and further subdivided based on phenotypes as multinucleated, micronucleated, split, or mixed error with ebryos displaying two different types of errors in each cell. All data collection and categorization was done retrospectively using the obtained time-lapse images to minimize inter- and intraobserver variability, by experienced embryologists blinded for the clinical outcomes.

### 2.4 Data analysis and statistics

Descriptive statistics were used to summarise clinical and laboratory variables. Continuous variables are presented as median with inter-quartile range (IQR), and categorical variables as frequencies with percentages.

Clinical outcomes included the number of positive pregnancy tests, used to calculate the pregnancy rate (PR); the number of observed fetal heartbeats on transvaginal ultra-sound, used to calculate the clinical pregnancy rate (CPR); and the number of embryo transfers resulting in live birth, used to calculate the live birth rate (LBR).

Associations between categorical variables were assessed using Fisher's exact test for 2 × 2 comparisons or the chi-square test for comparisons involving more than two groups. When expected cell counts were small or contingency tables exceeded 2 × 2, Fisher's exact test with Monte Carlo simulation was applied. *post hoc* pairwise testing was performed where appropriate.

The association between nuclear status at the two-cell stage (presence or absence of NE) and PR, CPR, and LBR was evaluated using contingency tables. Unadjusted odds ratios (ORs) with 95% confidence intervals (CIs) were calculated.

To assess embryo- and treatment-related predictors of NE, regression analyses were performed in the complete embryo cohort. Specifically:

1. Binary logistic regression was used with NE status as the dependent variable.
2. Ordinal logistic regression was used to evaluate ordered NE categories (no cell, one cell or two cells).
3. Multinomial logistic regression was applied to assess associations with specific NEP

All regression models included maternal age, paternal age, and treatment type as co-variates. Transfer type (fresh vs. frozen) was evaluated in preliminary analyses but was not included in the final models, as it was not significantly associated with NE or clinical outcomes. Results are presented as OR, proportional OR, or relative risk ratios (RRR) with corresponding 95% CI.

Because multiple embryo transfers originated from the same patient couple, additional sensitivity analyses were performed using mixed-effects logistic regression models to account for within-patient clustering. PR, CPR, and LBR were analysed as dependent variables, with patient identity included as a random intercept. Fixed effects included maternal age, paternal age, fertilization method, transfer type (fresh vs. frozen, where applicable), and nuclear error phenotype. Analyses were performed separately for cleavage-stage embryos, day 5 blastocysts, and day 6 blastocysts. Adjusted ORs with 95% CIs are reported.

Associations between NEPs and blastocyst quality were analysed using contingency-table methods. Blastocysts were categorised as top-quality blastocysts (TQB), good-quality blastocysts (GQB), or low-quality blastocysts (LQB). The TQB group included blastocysts with an A in either inner cell mass (ICM), trophectoderm (TE), or both, and expansion grade ≥4 on day 5 and ≥5 on day 6. The GQB group included 3AA, 3AB, and 3BA blastocysts on day 5, as well as all BB blastocysts with expansion grade ≥3 on day 5 or day 6. The LQB group included all blastocysts with a C in either ICM and/or TE, as well as all blastocysts with expansion grade 0–2.

Global differences in blastocyst quality distribution across NEP groups were assessed using Fisher's exact test for r × c contingency tables with Monte Carlo simulation. When a significant global association was observed, *post hoc* comparisons were performed by comparing each NEP group with embryos without NE using Fisher's exact test.

To explore the direction of observed associations, additional 2 × 2 analyses were conducted in which blastocyst quality was dichotomised as LQB vs. all other quality categories or TQB vs. all other categories. Unadjusted OR with 95% CI were calculated, and absolute differences in proportions [Δ percentage points (pp)] were reported to aid clinical interpretation. Adjustment for multiple testing in *post hoc* analyses was performed using the Benjamini–Hochberg false discovery rate procedure.

All statistical analyses were performed using a custom script in RStudio (version 2025.05.01) using R (version 4.4.1). A two-sided *p*-value < 0.05 was considered statistically significant.

## 3 Results

### 3.1 Cohort characteristics and overall clinical outcomes

The cohort consisted of 3,793 transferred embryos, from 2,420 ART cycles and 1,807 patient couples. The total number of transferred embryos per couple ranged from 1 to 10. Maternal age at oocyte retrieval was 32 years [IQR: 7, range 20–42], and paternal age at oocyte retrieval was 35 years [IQR: 8, range 21–58], maternal BMI was 25.5 kg/m^2^ [IQR: 6.3, range 15.5–38.8].

The clinical outcomes are presented in Table 1. Day 5 blastocysts resulted in significantly better -stage embryos and day 6 blastocysts. ICSI was associated with higher PR, CPR, and LBR compared with IVF.

**Table 1:** Clinical outcomes for complete cohort and divided based on developmental age and fertilization methods.

Cohort	Number (*n*)	PR (%)	CPR (%)	LBR (%)
Total cohort	3,793	50.9	43.4	37.7
Developmental age				
Cleavage stage	1,102	43.7^a^	37.8^a^	32.4^a^
Day 5 Blastocysts	2,273	54.2	46.3^b^	40.3
Day 6 Blastocysts	418	41.1^c^	33.0^c^	29.2^c^
Fertilization method				
ICSI	1,813	51.9	44.4	39.1
IVF	1,980	47.7*	40.5*	34.8*

PR, pregnancy rate; CPR, clinical pregnancy rate; LBR, live birth rate. Developmental age: Different superscript letters (a, b, c) within a column indicate statistically significant differences between all developmental stages (*p* < 0.05, chi-square test with *post hoc* pairwise comparisons). Fertilisation method.

* indicates *p* < 0.05 compared with ICSI (Fisher's exact test).

### 3.2 Nuclear errors in the transferred embryos

NE were observed in 20.8% (*n* = 229) of the transferred cleavage stages embryos, in 23.7% (*n* = 539) of the day 5 blastocysts, and in 28.9% (*n* = 121) of the day 6 blastocysts. Number of affected cells and NEP distributions are presented in Table 2.

**Table 2:** Frequency and distribution of nuclear errors in transferred embryos, observed in the two-cell embryo. Data are presented overall, but also by number of affected cells and by phenotypes.

Nuclear errors	Cleavage stage *n* (%)	Day 5 blastocysts *n* (%)	Day 6 blastocysts *n* (%)
Nuclear error, overall	229 (20.7)	539 (23.7)	121 (28.9)
Number of affected cells			
One	153 (13.9)	307 (13.5)	61 (14.4)
Two	76 (6.9)	232 (10.2)	60 (14.4)
Nuclear Error Phenotype			
Micronucleation	113 (10.3)	180 (7.9)	41 (9.8)
Multinucleation	69 (6.3)	178 (7.8)	32 (7.7)
Split nuclei	14 (1.3)	65 (2.9)	19 (4.5)
Mixed error	33 (3.0)	116 (5.1)	29 (6.9)

Across the cohort, neither maternal nor paternal age showed any association with the overall occurrence of NE. In the binary logistic model, maternal age was not associated with the presence of a NE (OR = 1.00, 95% CI 0.98–1.02), and paternal age demonstrated only a very small statistical effect (OR = 1.02, 95% CI 1.00–1.03, *p* = 0.035), an effect size too limited to indicate practical relevance. The fertilization method showed a clear and consistent association, embryos derived from ICSI had higher odds of exhibiting any NE compared with IVF (OR = 1.74, 95% CI 1.49–2.03, *p* < 0.001).

In the ordinal logistic regression assessing error severity, defined as the number of affected blastomeres, maternal age again showed no association, while paternal age displayed a small increase in proportional odds (OR = 1.02, 95% CI 1.00–1.03, *p* = 0.019), although again the magnitude of the effect remained minimal. ICSI was consistently associated with higher nuclear error class in this model as well (OR = 1.77, 95% CI 1.52–2.06, *p* < .001)

In the multinomial logistic model, fertilization method showed distinct phenotype specific associations. Compared with embryos displaying normal nuclear morphology, embryos fertilized by ICSI had increased relative risk of multinucleation (RRR = 2.00, 95% CI 1.55–2.59), micronucleation (RRR = 1.29, 95% CI 1.03–1.62), split nuclei (RRR = 2.04, 95% CI 1.34–3.10), and mixed errors (RRR = 2.25, 95% CI 1.63–3.10).

### 3.3 Impact of nuclear errors on clinical outcomes

In the pooled cohort, embryos exhibiting NE showed lower PR than embryos without NE (PR 46.3% vs. 50.8%, *p* < 0.05) while CPR and LBR did not differ significantly. However, outcomes varied markedly by developmental stage, indicating stage-dependent effects.

#### 3.3.1 Cleavage-stage embryos

Among embryos transferred at cleavage-stage, the presence of NE was associated with significantly reduced clinical outcomes across all endpoints. Embryos with NE had reduced PR, CPR, and LBR compared with embryos without NE (PR 33.2% vs. 46.5%, CPR 27.5% vs. 40.5%, LBR 23.2% vs. 34.8%, *p* < 0.001).

When outcomes were stratified by the number of affected blastomeres, a clear pattern emerged. Embryos with a single affected cell showed significantly lower PR (35.9%), CPR (30.0%), and LBR (26.1%) compared with embryo without NE (*p* < 0.05), while embryos with errors in both blastomeres had the poorest outcomes (PR 27.6%, CPR 22.4%, LBR 17.3%, *p* < 0.01).

Analysis by phenotype demonstrated further heterogeneity (global *p*-values: PR = 0.001; CPR = 0.004; LBR = 0.006). Micronucleation was consistently associated with reduced outcomes across all endpoints relative to embryos without NE (PR 31.8%, CPR 26.5%, LBR 22.1%, *p* < 0.01). Split nucleation showed markedly low rates (PR and CPR 14.3%, LBR 7.1%, *p* < 0.05), although sample sizes were small (*n* = 14). Mixed error phenotypes also demonstrated significantly lower PR (27.3%, *p* < 0.05), whereas CPR (24.2%) and LBR (21.2%) differences did not reach statistical significance. Multinucleation yielded outcome rates comparable to those of embryos without NE across all endpoints (PR 42.2%, CPR 33.3%, LBR 29.4%).

**Figure 1:**
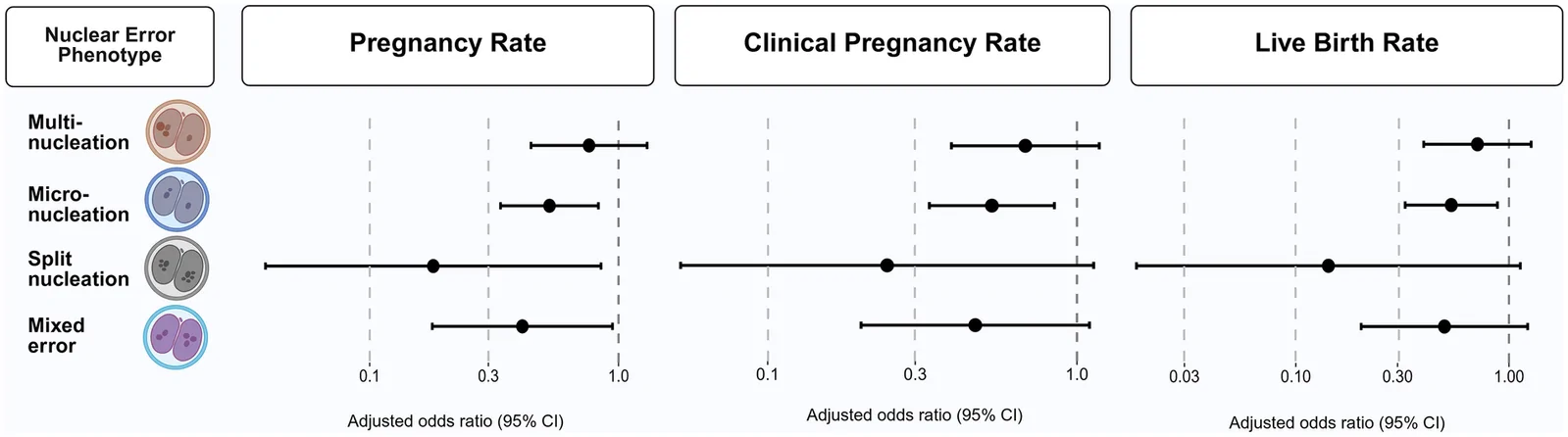
Association between nuclear error phenotype and clinical outcomes following cleavage-stage embryo transfer. Adjusted odds ratios (ORs) and 95% confidence intervals (CIs) for pregnancy rate (PR), clinical pregnancy rate (CPR), and live birth rate (LBR) according to nuclear error phenotype observed at the two-cell stage. Odds ratios are presented relative to embryos without nuclear errors (reference category, OR = 1). Estimates were derived from mixed-effects logistic regression models adjusted for maternal age, paternal age, fertilization method, and patient-level clustering. Patient identity was included as a random intercept to account for repeated embryo transfers from the same patient. Created in BioRender. Adolfsson, E. (2026) https://BioRender.com/ura910m.

To account for repeated transfers from the same patient and potential confounding factors, additional mixed-effects logistic regression analyses were performed (Table 3). Among cleavage-stage embryos, micronucleation remained independently associated with reduced PR (OR = 0.53, 95% CI 0.34–0.83, *p* = 0.006), CPR (OR = 0.53, 95% CI 0.33–0.85, *p* = 0.008), and LBR (OR = 0.54, 95% CI 0.33–0.88, *p* = 0.014). Split nuclei were associated with markedly reduced odds of PR (OR = 0.18, 95% CI 0.04–0.85, *p* = 0.030), with similar effect estimates observed for CPR (OR = 0.24) and LBR (OR = 0.14), although confidence intervals were wide because of the limited number of affected embryos (*n* = 14). Mixed error phenotypes were associated with reduced odds of PR (OR = 0.41, 95% CI 0.18–0.95, *p* = 0.036), whereas multinucleation was not independently associated with outcome. These associations remained after adjustment for maternal age, paternal age, fertilization method, and patient-level clustering. See Figure 1.

**Table 3:** Adjusted mixed-effects logistic regression analysis of cleavage-stage embryos. Data is presented as adjusted odds ratio with 95% confidence interval.

Nuclear error phenotype	PR (OR)	PR, p	CPR (OR)	CPR, p	LBR (OR)	LBR, p
Multinucleation	0.76 (0.44–1.30)	0.320	0.68 (0.39–1.18)	0.171	0.71 (0.40–1.27)	0.249
Micronucleation	0.53 (0.34–0.83)	0.006	0.53 (0.33–0.85)	0.008	0.54 (0.33–0.88)	0.014
Split nuclei	0.18 (0.04–0.85)	0.030	0.24 (0.05–1.13)	0.072	0.14 (0.02–1.13)	0.065
Mixed error	0.41 (0.18–0.95)	0.036	0.47 (0.20–1.10)	0.081	0.50 (0.20–1.22)	0.128

PR = pregnancy rate; CPR = clinical pregnancy rate; LBR = live birth rate; CI = confidence interval (95%).

Odds ratios are presented relative to embryos without nuclear errors (reference category). Estimates were derived from mixed-effects logistic regression models adjusted for maternal age, paternal age, fertilization method, and patient-level clustering. Patient couple identity was included as a random intercept to account for repeated embryo transfers to the same patient/patient couples.

#### 3.3.2 Blastocysts

For blastocyst transfers, no significant differences were observed by NE presence, NE1/NE2 class, or NE phenotype. Day 5 rates were essentially indistinguishable between NE and no NE across PR (53.1% vs. 54.5%; *p* = 0.55), CPR (46.2% vs. 46.3%; *p* = 1), and LBR (40.8% vs. 40.3%; *p* = 0.88); global tests for NE1/NE2 and for phenotypes were likewise non-significant (*p*-values ≥ 0.52). Day 6 rates displayed the same pattern for NE and no NE: PR 41.1% vs. 41.3%; *p* = 1, CPR 32.7% vs. 33.9%; *p* = 0.82, and LBR 29.0% vs. 30.3%; *p* = 0.90; global tests for NE1/NE2 and for phenotypes were non-significant (*p*-values ≥ 0.10).

Mixed-effects logistic regression analyses yielded similar findings. No independent associations between NEP and PR, CPR, or LBR were observed among transferred day 5 blastocysts, with adjusted OR estimates generally close to unity. Likewise, no significant associations were observed among day 6 blastocysts, although confidence intervals were wider because of the smaller sample size in this subgroup.

### 3.4 Correlation between blastocyst quality and nuclear error phenotypes

In the pooled cohort, the presence of NE as a binary variable was significantly associated with changes in blastocyst quality distribution (GQB, LQB, TQB), *p* = 0.037. When embryos were stratified by NEP, a significant association with blastocyst quality emerged (*p* = 0.0037), indicating heterogeneity across specific nuclear error types. See Table 4.

**Table 4:** Association between nuclear error phenotype and blastocyst quality. Distribution of blastocyst quality according to nuclear error phenotype (NEP) observed at the two-cell stage.

Nuclear status	GQB	LQB	TQB	Global *p*-value	Quality Shift
	*n* (%)	*n* (%)	*n* (%)		
No nuclear errors	649 (31.7)	124 (6.1)	1,270 (62.5)	Ref	-
Multinucleation	63 (30.0)	8 (3.8)	139 (66.2)	0.361	-
Micronucleation	82 (37.1)	19 (8.6)	120 (54.3)	0.078^a^	↓ TQB (−8.2 pp)
					↑ LQB (+2.5 pp)
Split nuclei	32 (38.1)	11 (13.1)	41 (48.8)	0.037^b^	↓ TQB (−13.7 pp)
					↑ LQB (+7.0 pp)
Mixed error	53 (36.6)	14 (9.7)	78 (53.8)	0.078^a^	↓ TQB (−8.7 pp)
					↑ LQB (+3.6 pp)

GQB, good-quality blastocyst; LQB, low-quality blastocyst; TQB, top-quality blastocyst; pp, percentage points. Global *p*-values derived from Fisher's exact test (r × c) with Monte Carlo simulation. *post hoc* comparisons vs. embryos without nuclear errors were adjusted using the Benjamini–Hochberg procedure.

a Borderline significance after adjustment.

b Statistically significant after adjustment.

Post hoc comparisons demonstrated that blastocysts with split nuclei showed a significantly different quality distribution compared with embryos without NE (*p* adj. = 0.037), whereas differences for micronucleation and mixed phenotypes did not re-main statistically significant after correction. Blastocysts with multinucleation exhibited a quality distribution comparable to that of blastocysts without NE.

To further characterise the direction of these associations, blastocyst quality was dichotomised for additional analyses. Embryos with split nuclei showed an increased pro-portion of low-quality blastocysts compared with embryos without NE (LQB 13.1% vs. 6.1%; Δ = +7.0 pp, OR 0.43 [0.22–0.93]), accompanied by a reduced proportion of top-quality blastocysts (TQB 48.8% vs. 62.5%; Δ = −13.7 pp, OR 0.57 [0.36–0.91]).

Micronucleation and mixed error phenotypes showed similar but weaker patterns. For micronucleation, the proportion of low-quality blastocysts was modestly increased (8.6% vs. 6.1%; Δ = +2.5 pp, OR 0.69 [0.41–1.21]), alongside a reduction in top-quality blastocysts (54.3% vs. 62.5%; Δ = −8.2 pp, OR 0.71 [0.53–0.95]). Mixed error phenotypes showed comparable shifts, with an increased LQB proportion (9.7% vs. 6.1%; Δ = +3.6 pp, OR 0.61 [0.34–1.18]) and reduced TQB proportion (53.8% vs. 62.5%; Δ = −8.7 pp, OR 0.70 [0.49–0.99]). In contrast, multinucleation was not associated with impaired embryo quality, with a lower proportion of low-quality blastocysts (3.8% vs. 6.1%; Δ = −2.3 pp, OR 1.64 [0.79–3.94]) and no evidence of depletion in top-quality blastocysts (66.2% vs. 62.5%; Δ = +3.7 pp, OR 1.17 [0.86–1.61]).

Overall, these findings indicate that the association between NE and blastocyst quality is phenotype-specific. In particular, split errors were associated with a shift away from top quality morphology towards lower-quality classifications, whereas other nuclear error phenotypes demonstrated limited or no impact on embryo quality.

## 4 Discussion

In this study, we characterised the clinical relevance of NE observed at the two-cell stage, with a specific focus on phenotype–dependent effects on embryo development, blastocyst quality, and clinical outcomes following single embryo transfer. By integrating detailed time-lapse-based nuclear annotation with stage-specific outcome analysis in a large cohort, we demonstrate that the impact of NE is strongly dependent on both developmental stage at transfer and NEP.

A central observation is that NEP represent transient historical events at the two-cell stage rather than persistent morphological abnormalities. Nevertheless, all phenotypes remained detectable among transferred blastocysts, indicating that NE are not uniformly lethal. Importantly, the relative distribution of phenotypes changed across developmental stages. Split nuclei increased in proportion from cleavage-stage embryos (1.3%) to day 5 blastocysts (2.9%), and further to day 6 blastocysts (4.5%). In contrast, the frequencies of micronucleation and multinucleation remained comparatively stable across stages. These shifts suggest delayed developmental progression and cumulative clinical selection for embryos exhibiting the most overtly abnormal nuclear patterns.

These developmental dynamics were mirrored by the stage-specific clinical outcome data. At the cleavage stage, the presence of NE was associated with a pronounced reduction across all clinical endpoints, with PR decreasing from 46.5% to 33.2%, CPR from 40.5% to 27.5%, and LBR from 34.8% to 23.2%. Stratification by the number of affected blastomeres revealed a pattern where embryos affected in both blastomeres showed the poorest outcomes. Phenotype-specific analyses further demonstrated marked heterogeneity: micronucleation was consistently associated with reduced clinical outcomes, while split nuclei showed extremely low outcomes, including a LBR of 7.1%. In contrast, multi-nucleation at the cleavage stage yielded outcome rates comparable to embryos without NE. These findings were supported by mixed-effects logistic regression analyses accounting for maternal age, paternal age, fertilization method and repeated transfers from the same patient. In these adjusted models, micronucleation remained independently associated with reduced PR, CPR and LBR, while split nuclei and mixed error phenotypes also showed adverse associations with reproductive outcome. In contrast, multinucleation was not independently associated with any clinical endpoint. The consistency between unadjusted and adjusted analyses suggests that the observed phenotype-specific effects are not solely explained by differences in patient characteristics or treatment factors.

Among blastocyst transfers, no differences in PR, CPR, or LBR were observed between embryos with and without NE, regardless of phenotype or the number of affected blastomeres. Clinical outcomes were essentially indistinguishable, and global tests across NE classes and phenotypes were uniformly non-significant. These findings indicate that the adverse associations observed at the cleavage stage are no longer detectable among transferred blastocysts. One possible explanation is that embryos carrying the most severe consequences of early nuclear abnormalities fail to achieve blastocyst development or are not selected for transfer because of inferior morphology. In this context, extended culture may facilitate developmental and morphological selection against embryos with the greatest developmental impairment. Although a biological correction of early abnormalities cannot be excluded, the present study was not designed to distinguish between correction of nuclear abnormalities and selective loss of affected embryos.

Analysis of blastocyst quality demonstrated that NE analysed as a binary variable were not significantly associated with quality distribution. However, phenotype-specific stratification revealed clear heterogeneity, driven primarily by split nuclei. Embryos with split nuclei demonstrated a shift away from top-quality blastocysts, with a reduction in TQB proportions from 62.5% to 48.8% and a concomitant increase in low-quality blastocysts from 6.1% to 13.1%. In contrast, multinucleation was not associated with impaired blastocyst quality, and micronucleation and mixed errors showed only modest, non-significant shifts after correction for multiple testing. These findings indicate that the adverse associations observed at the cleavage stage are no longer detectable among transferred blastocysts. The absence of both unadjusted and adjusted associations in day 5 blastocysts suggests that extended culture and subsequent embryo selection substantially reduce the clinical relevance of early nuclear abnormalities among embryos ultimately selected for transfer.

Several previous studies have examined nuclear abnormalities at the two-cell stage in relation to clinical outcomes, including live birth, but direct comparison is limited by differences in phenotype resolution and study design. Sayed et al. (17). reported an association between nucleation errors at the two-cell stage and reduced live birth rates, treating nuclear abnormalities largely as a binary variable and including mixed transfer strategies without phenotype-specific stratification. Studies examining multinucleation at the two-cell stage have reported heterogeneous clinical outcomes, largely reflecting differences in study design and level of nuclear resolution. Desch et al (31). reported significantly reduced implantation and LBR in ICSI cycles when multinucleation was present at the two-cell stage, particularly when both blastomeres were affected, based on cleavage-stage assessment and without extended phenotype stratification. In contrast, Talbot et al., analysing single blastocyst transfers, demonstrated favourable PR and LBR outcomes for bi-nucleated embryos, while true multinucleated embryos showed reduced clinical outcomes compared with mononucleated embryos (18). Importantly, neither study differentiated multinucleation from other abnormal nuclear configurations such as micronucleation or split nuclei. The present findings suggest that some of the apparent discrepancies in the literature may be explained by unrecognised phenotype heterogeneity and by differences in developmental stage at which embryos were selected for transfer. In contrast, the present study resolves NE into distinct phenotypes and demonstrates that adverse associations with live birth are driven primarily by split nuclei, whereas multinucleation and bi-nucleation exhibit largely neutral behaviour following blastocyst selection.

From a clinical perspective, the present data indicate that survival through the cleavage stage constitutes an important developmental bottleneck for embryos affected by nuclear errors. The absence of outcome differences among transferred blastocysts may reflect developmental and clinical selection against embryos carrying the most severe consequences of early nuclear abnormalities. Consistent with this interpretation, split nuclei were associated with reduced blastocyst quality and became increasingly enriched among later developmental stages. Therefore, the apparent loss of prognostic value at the blastocyst stage should not necessarily be interpreted as evidence that early abnormalities have been corrected, but rather that embryos exhibiting the greatest developmental impairment may be removed from the population before transfer.

The adjusted analyses further suggest that the clinical relevance of NE is phenotype-dependent. Micronucleation remained independently associated with reduced PR, CPR and LBR following cleavage-stage transfer, indicating that this phenotype carries prognostic information beyond established clinical variables. Split nuclei showed similarly adverse effect estimates, although confidence intervals were wider because of the limited number of affected embryos. In contrast, multinucleation demonstrated largely neutral behaviour across both unadjusted and adjusted analyses.

Apart from our previous analysis of blastocyst formation, no studies to date have examined phenotype-specific nuclear errors at the two-cell stage in relation to both blastocyst quality and live birth outcomes following single embryo transfer. Live birth represents the most clinically meaningful outcome in assisted reproduction, and the observation that phenotype-specific differences persist at this level underscores the long-term relevance of early nuclear events.

Additional strengths of this study include the large cohort of 3,793 single embryo transfers with clinical follow-up, enabling adequately powered analyses across develop-mental stages, nuclear error severity, and phenotype-specific subgroups. Restriction to single embryo transfer eliminates confounding from multiple embryo transfers and allows direct attribution of outcomes to the transferred embryo. Furthermore, the use of non-invasive time-lapse imaging enabled phenotype-specific annotation while remaining fully compatible with routine clinical practice, an important consideration in settings where preimplantation genetic testing is restricted or unavailable.

Several limitations should be considered. The retrospective, single-centre design may limit generalisability. Embryo selection was based on standard morphology, and embryologists were not blinded to nuclear status, introducing potential selection bias. Cleavage-stage embryos were not formally graded beyond transfer suitability, precluding assessment of interactions between cleavage-stage morphology and nuclear error phenotype. Data covers the span of 13 years, and although all embryo culture, selection, cryopreservation and transfer steps have remained identical over the years, small variations in environment, staffing etc., are inevitable. Although mixed-effects models were used to account for repeated transfers from the same patient, some phenotype-specific analyses, particularly those involving split nuclei, remained limited by small sample size.

## 5 Conclusions

When embryo selection is performed at the cleavage stage, incorporation of nuclear error phenotypes into embryo assessment may provide additional clinically relevant information. Both the number of affected blastomeres and the specific nuclear error phenotype were associated with reproductive outcome, and phenotype-specific associations persisted after adjustment for maternal age, paternal age, fertilization method, and patient-level clustering. Micronucleation showed the most consistent adverse association with pregnancy, clinical pregnancy, and live birth, while split nuclei were associated with particularly poor clinical outcomes, although the number of affected embryos was limited.

In contrast, when embryo selection is performed at the blastocyst stage, nuclear status at the two-cell stage was not associated with clinical outcomes. These findings suggest that extended culture may facilitate developmental selection against embryos with the most severe consequences of NE, thereby reducing the prognostic value of nuclear status among embryos ultimately selected for transfer.

## Data Availability

The raw data supporting the conclusions of this article will be made available by the authors, without undue reservation.
